# One-pot synthesis of amine-functionalized graphene oxide by microwave-assisted reactions: an outstanding alternative for supporting materials in supercapacitors

**DOI:** 10.1039/c7ra13514a

**Published:** 2018-02-07

**Authors:** C. C. Caliman, A. F. Mesquita, D. F. Cipriano, J. C. C. Freitas, A. A. C. Cotta, W. A. A. Macedo, A. O. Porto

**Affiliations:** Department of Chemistry, Federal University of Espírito Santo – UFES Vitória 29075-910 ES Brazil cristianocaliman@gmail.com; Laboratory of Carbon and Ceramic Materials, Department of Physics, Federal University of Espírito Santo – UFES Vitória 29075-910 ES Brazil; Nuclear Technology Development Center – CNEN-CDTN Belo Horizonte MG Brazil; Department of Chemistry, Federal University of Minas Gerais, UFMG Belo Horizonte 31270-901 MG Brazil

## Abstract

A simple and straightforward method using microwave-assisted reactions is presented for the functionalization of graphene oxide with aromatic and non-aromatic amines, notedly dibenzylamine (DBA), *p*-phenylenediamine (PPD), diisopropylamine (DPA) and piperidine (PA). The as-synthesized amine-functionalized graphene oxide materials (amine-GO) were characterized using spectroscopic techniques including XRD, FTIR, ^13^C NMR, XPS, TEM for imaging and thermogravimetric analysis (TGA). The characterization confirmed the functionalization for all amines, reaching relatively high surface nitrogen atomic concentrations of up to 8.8%. The investigations of electrochemical behavior for the amine-GOs show the significant improvement in GO's electrochemical properties through amine functionalization, exhibiting long life cycle stability and reaching specific capacitance values of up to 290 F g^−1^ and 260 F g^−1^ for GO-PA and GO-DPA samples, respectively, confirming their potential application as alternative supporting materials in supercapacitors.

## Abstract 2

A simple and straightforward method using microwave-assisted reactions is presented for the functionalization of graphene oxide with aromatic and non-aromatic amines.

## Graphical abstract

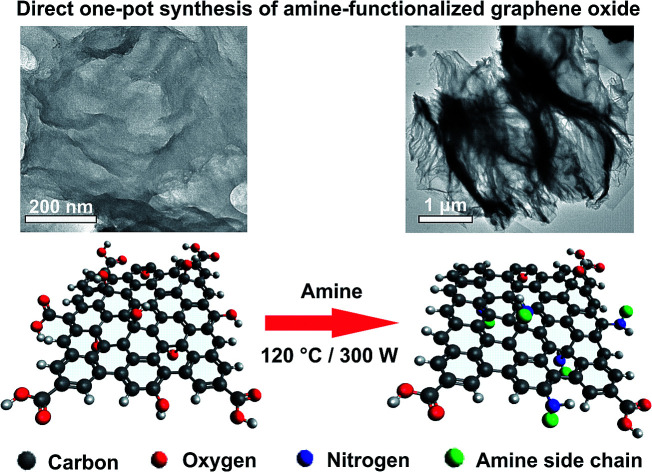

## 1. Introduction

The rapid development of the electronics industry and growing concerns about environmental issues has led to the research and development of materials for energy storage devices with high efficiency and performance. In this context, supercapacitors, which consist of intermediate systems between conventional (or dielectric) capacitors and batteries, are the best candidates due to their high energy storage capacity, low maintenance costs, fast charge–discharge processes and long cycle life.^1^ Generally, supercapacitors are classified into two types, according to the energy storage mechanism: pseudocapacitors and electric double-layer capacitors. Pseudocapacitors, whose working mechanism involves the occurrence of reversible redox reactions on the electrodes surface, offer greater specific capacitance than electric double-layer capacitors.^2^

Most solid-state pseudocapacitors are based on materials such as transition metal oxides and hydroxides and composite materials with excellent properties such as being non-polluting, having good charge density, fast charge–discharge and long cycle life.^3^ As an example, some interesting materials based on metal–organic frameworks (MOFs) have been used as precursors to prepare flexible asymmetric supercapacitors such as Co_3_O_4_ and N-doped carbon nanosheets^4^ and hollow NiCo_2_O_4_ arrays.^5^ More recently electrodes of CoFe_2_O_4_/NiFe_2_O_4_ nanocomposites have been prepared presenting high specific capacitance of 269 F g^−1^ and good electrochemical stability.^6^ However, such materials generally exhibit low electrical conductivity. In order to bypass such limitations, pseudocapacitive materials are normally supported on carbon-based nanomaterials such as carbon nanotubes and graphene.

Graphene oxide (GO), an oxygen-containing derivative of graphene, has been widely studied as a direct precursor for this material as well as a platform for a plethora of applications such as lithium-ion batteries,^7–9^ solar cells,^10–12^ supercapacitors,^13,14^ biological systems^15–17^ and water desalination.^18,19^ The presence of different oxygen functional groups in GO allows a higher interaction with polar solvents (*e.g.*, water) and provides different sites for further chemical functionalization.

It is well known that nitrogen atoms in amine groups are more nucleophilic than oxygen atoms,^20^ so several studies of amine-functionalization of the GO structure have been carried out in order to increase its interfacial binding to materials of interest. For example, amines of different chain lengths were applied for functionalization of GO and promoted its covalent integration into epoxy resin matrices through chemical reactions between the amine and epoxy groups.^21^ Moreover, many amine types have been applied for reinforcement of epoxy resins,^22^ selective dye adsorption,^23^ polymer composites,^24,25^ carbon dioxide capture,^26,27^ electrochemical detectors^28^ and catalysis,^29,30^ among many others. In this context, the microwave technique has been shown to be very advantageous for functionalization of carbon nanostructures. In microwaves the heating is generated in the interior of the sample and transferred outwards during the process, while in conventional heating this transference happens in the opposite way. Therefore this technique offers a series of advantages like higher reaction yields, rapidity and an easier work up of the reaction products.^31^

The use of amine-functionalized GO in supercapacitors has also been thoroughly investigated. Mostly the GO structure is either grafted or covalently bonded to polyaniline,^32,33^*p*-phenylenediamine^34,35^ and other amine types and then chemically or thermally reduced to eliminate the oxygen groups. In the present study, four different amines (*p*-phenylenediamine, dibenzylamine, diisopropylamine and piperidine) have been applied in a one-pot synthesis of amine-functionalized reduced graphene oxide, using a microwave-assisted route, and their electrochemical performance was investigated.

## 2. Experimental section

### 2.1 Materials

Natural graphite flakes were purchased from Nacional de Grafite Ltda company (Minas Gerais, Brazil). Phosphoric acid (H_3_PO_4_, >99%), sulfuric acid (H_2_SO_4_, 95–98%), dimethylformamide (DMF), *p*-phenylenediamine (>99%), dibenzylamine (97%), diisopropylamine (99%) and piperidine (99%) were all purchased from Sigma-Aldrich; potassium permanganate (KMnO_4_, 99%) was acquired from Neon Commercial.

### 2.2 Synthesis

GO was prepared by the route first described by Marcano *et al*.^36^ In a typical experiment, KMnO_4_ (18 g) was added to 3 g of natural graphite flakes and then dispersed into a 400 mL solution of H_2_SO_4_/H_3_PO_4_ (9 : 1 molar ratio). The mixture was stirred at 50 °C for 12 h. After this, the mixture was cooled down to room temperature and poured onto 400 mL of ice and 3 mL of H_2_O_2_ aqueous solution 30% v/v. The resulting GO suspension was filtered and then centrifuged (6000 rpm, 1 h). The obtained solid was washed with distilled water, HCl aqueous solution (30%, v/v) and ethanol. The obtained GO was then dried overnight at 100 °C.

For the synthesis of the GO-amine materials the following amines were used: *p*-phenylenediamine (PPD), dibenzylamine (DBA), diisopropylamine (DPA) and piperidine (PA). GO (200 mg) was exfoliated in 50 mL dimethylformamide (DMF) within ultrasonic bath during 20 min and then 30 mL amine was added. The suspension was sonicated for 20 min. The resulting mixture was heated in a microwave reactor up to 120 °C with a heating rate of 10 °C min^−1^ and the system was kept at this temperature during 30 min. 300 mL anhydrous ethanol was added to the obtained suspension and finally the functionalized GO was separated by centrifugation and washed with distilled water and ethanol. The obtained GO-amine was vacuum-dried at 100 °C for 6 h. Four GO-amine samples were obtained and named according to the amine molecule: GO-PPD, GO-DBA, GO-DPA and GO-PA.

### 2.3 Characterization

FTIR spectra were recorded at room temperature with a spectrometer Perkin Elmer SPECTRUM 400. Powder X-ray diffraction (XRD) analyses were carried out in a Bruker D8 DISCOVER equipment operating with Cu-Kα radiation (1.5406 Å) generated at 40 kV and 40 mA with 2*θ* from 5 to 90°. Thermogravimetry (TG) curves were obtained in a Shimadzu TGA 50/50H equipment under argon flux (50 mL min^−1^) and heating rate of 10 °C min^−1^ up to 600 °C. X-ray photoelectron spectroscopy (XPS) measurements were carried out in ultrahigh vacuum chamber (base pressure lower than 2.0 × 10^−9^ mbar) using monochromatic Al-Kα (1486.6 eV) excitation source with output power set at 350 W and SPECS hemispherical electron energy analyzer PHOIBOS 150 MCD. Survey and high-resolution spectra were recorded with band pass energies of 50 and 40 eV, respectively. The scans were acquired using a flood gun emission current of 0.8 μA for a charge compensation. CasaXPS software (v 2.3.15) was used to analyze all XPS data (http://www.casaxps.com). Transmission electron microscopy (TEM) images were obtained in an Electron Microscope JEOL JEM-1400 with 800 000× magnification and 120 kV. For these analyses the samples were dispersed in ethanol in ultrasonic bath and dripped in a formvar film on 200 mesh copper grid. ^13^C nuclear magnetic resonance (NMR) spectra were recorded in a Varian-Agilent 400 MHz spectrometer, operating at 100.52 MHz (corresponding to a magnetic field of 9.4 T). A triple resonance radiofrequency (RF) probehead with 4 mm diameter zirconia rotors was used for experiments with magic angle spinning (MAS) at 14 kHz spinning frequency. ^13^C NMR signal of methyl groups in hexamethylbenzene (HMB) – with a chemical shift of 17.3 ppm with respect to tetramethylsilane (TMS) – was used as a the secondary chemical shift reference. A pulse sequence specially designed to avoid probe background was used with a π/2 pulse (4.3 μs) immediately followed by a pair of π pulses (8.6 μs) and detection of the free induction decay (FID).^37^ The spectra were obtained by FIDs Fourier transform after 3000 scans accumulation with 15 s interval, 250 kHz spectral window and 8.192 ms acquisition time. The Brunauer–Emmett–Teller (BET) surface area, Barret–Joyer–Halenda (BJH) pore size distributions for the different materials were obtained by nitrogen adsorption using AutosorbQ surface area and porosity analyzer (Quantachrome, USA). Prior to the analysis the samples were outgassed at 80 °C for 24 hours under vacuum conditions.

### 2.4 Electrode preparation and electrochemical characterization

All electrochemical measurements were performed in AUTOLAB PGSTAT 302N equipment. The electrodes were prepared by dispersing 1.0 mg of each sample in 190 μL DMF and 10 μL Nafion® solution in ultrasonic bath for 5 min to form a homogeneous paste. Each dispersion (10 μL) was deposited onto a glassy carbon electrode (diameter 4 mm). Next the electrodes were dried in an oven at 120 °C for 1 hour. For all CV and galvanostatic charge–discharge experiments a three electrode system was applied using a potential range of 0.0–1.0 V. The values of specific capacitance (*C*) were calculated as described in the literature^38^ using the relationship *C* = (*I* × *t*)/(*V* × *m*) where, *I* is the constant discharge current, *t* represents the discharge time, *V* is the discharging voltage and *m* is the mass of active material deposited on the electrode. Electrochemical impedance spectroscopy analyses were carried out using a sinusoidal signal of 10 mV and a frequency range from 100 kHz to 1 Hz.

## 3. Results and discussion

### 3.1 Characterization

**Fig. 1:**
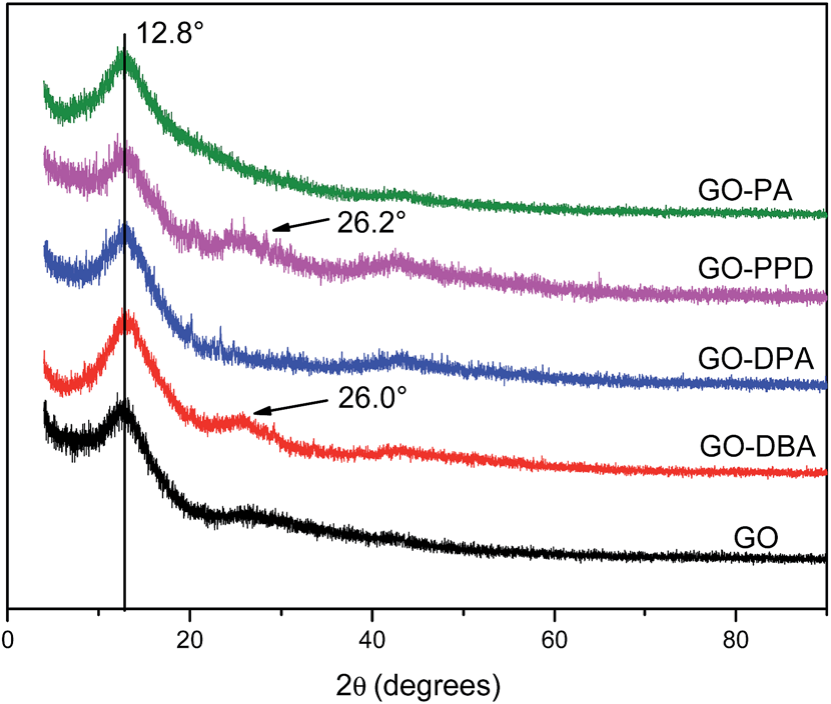
XRD patterns of GO, GO-DBA, GO-DPA,GO-PPD and GO-PA.

The XRD pattern is a very important tool to study the changes in the inter-sheet gap of GO materials.^39^ The introduction of alkylamine groups in the GO structure affects its *d*-spacing and thereby the degree of GO stacking. XRD patterns, Fig. 1, show typical graphene oxide diffraction peaks at 2*θ* = 12.8°, associated to the (002) diffraction planes, an interlayer spacing of 0.69 nm, calculated from Bragg's Law.^40^ For natural graphite the main peak is located at 2*θ* = 26° and the shift of this peak to 2*θ* = 12.8° occurs due to the expansion in the interlayer separation caused by the introduction of functional groups and the intercalation of water molecules between the graphene-like layers after oxidation.^40,41^

The same peak observed for GO at 2*θ* = 12.8° was also observed for all amine functionalized materials, indicating that the amine groups were not inserted between the layers. However, for the GO-PPD, GO-DPA and GO-DBA materials, Fig. 1, a slight shift in the peak position to higher angles has been observed, to 2*θ* = 13.2° (interlayer spacing = 0.66 nm), as well as the appearance of low intensity peaks at 2*θ* = 26.2° and 26.1° for GO-PPD and GO-DBA, respectively. These findings possibly indicate that a partial reduction of the GO structures may have occurred, decreasing the number of functional groups at the interlayer spaces and the appearance of a diffraction peak typical of disordered carbon materials. Thus, the introduction of the amine groups into the GO structure seems to be accompanied by a partial reduction of the other functional groups, with the interlayer spacing remaining practically unchanged.

**Fig. 2:**
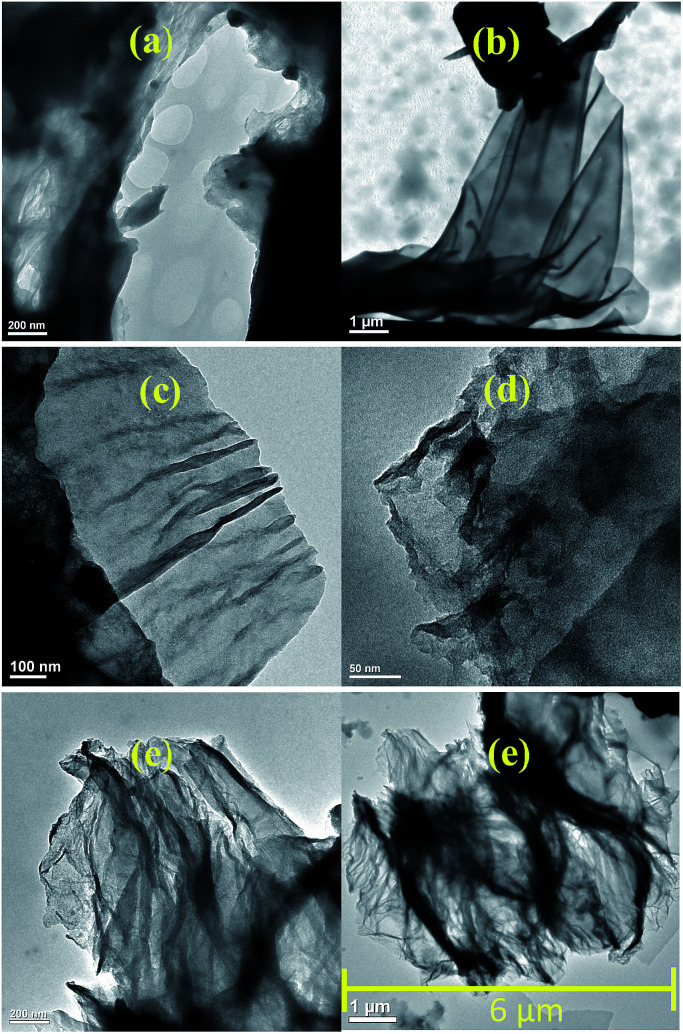
TEM images for GO (a), GO-DBA (b), GO-DPA (c), GO-PA (d) and GO-PPD (e).

TEM images, Fig. 2, showed that the amine-GO structures present a lower degree of aggregation and packing of the functionalized materials nanosheets as evidenced for GO-PPD, Fig. 2e, compared to GO, Fig. 2a. These results are an indicative of the amine functionalization effectiveness despite the materials interlayer distance having remained the same as before the functionalization reactions. In another study, in which graphene oxide was functionalized with diethylenetriamine,^42^ similar structures were found, also observing a greater number of curvatures and surface folds in the two-dimensional structures functionalized with amines when compared with non-functionalized GO. These curvatures were associated with the more regular hydrogen bonds (less random than in the GO structure) between the amine molecules. Such a characteristic surface wrinkling would be an important advantage for applications in catalytic reactions, due to the higher accessibility of the catalytic sites.

**Fig. 3:**
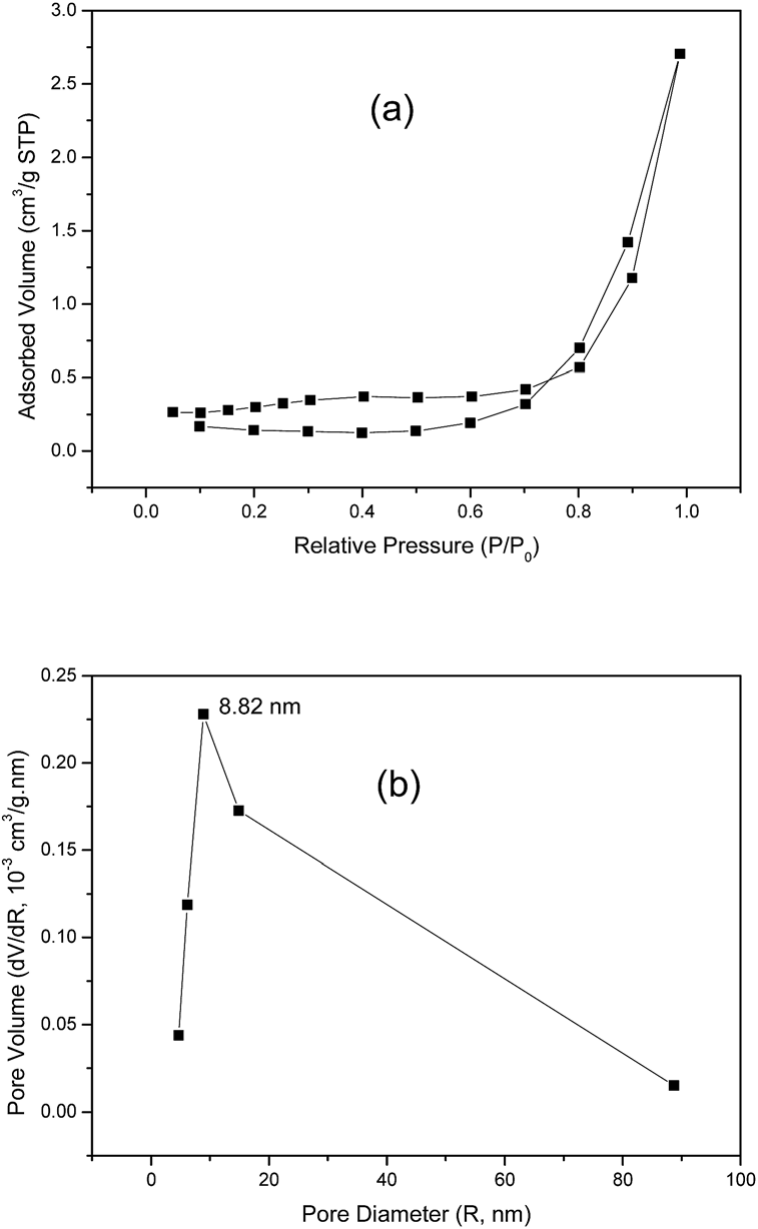
N_2_ adsorption–desorption isotherm for GO: (a) BET surface area measurement and (b) BJH pore size distribution.

**Fig. 4:**
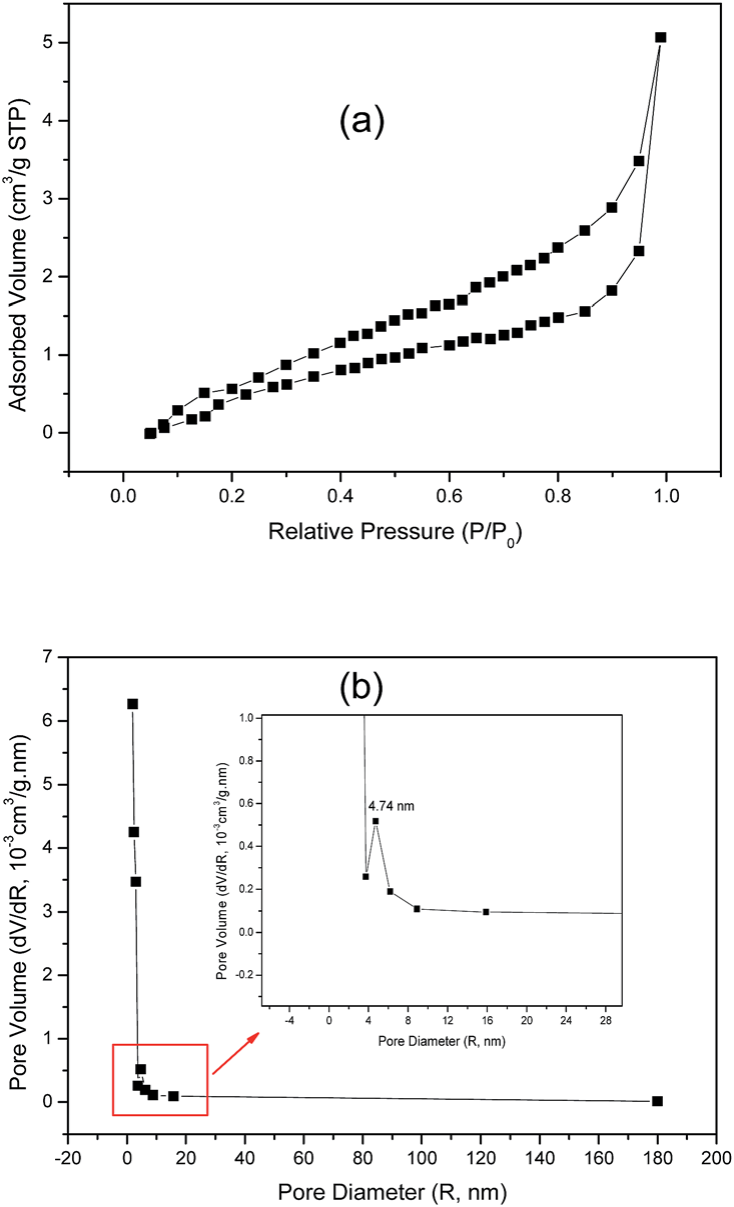
N_2_ adsorption–desorption isotherm for GO-DBA: (a) BET surface area measurement and (b) BJH pore size distribution.

The specific surface area and pore-size distribution for GO and the functionalized materials were measured using the nitrogen adsorption–desorption technique and the isotherms for GO and GO-DBA are presented in Fig. 3 and 4. For GO (Fig. 3a) the isotherm exhibits the combined characteristics of type-III and type-V isotherms, according to IUPAC classification,^43^ while for GO-DBA (Fig. 4a) it shows a combination of type-II and type-IV isotherms. For all materials the surface areas were found to be smaller than 10 m^2^ g^−1^, which could be expected for hydrothermally reduced graphene oxide materials that normally exhibit relatively low values of specific surface area due to intense agglomeration of rGO flakes during the vacuum drying process.^44,45^ An alternative to increase the surface area avoiding the aggregation could be the freeze drying.^46^ However, since the amine-GO materials are exfoliated and deposited on a conductive polymeric matrix during electrode preparation, this drawback should be mitigated. Furthermore, the pore volume distribution curves (Fig. 3b and 4b) obtained using BJH method indicated the presence of mesoporous structures for both GO and functionalized GO, showing maximum pore volumes for pore diameters of 8.82 nm for GO and 4.74 nm for GO-DBA.

**Fig. 5:**
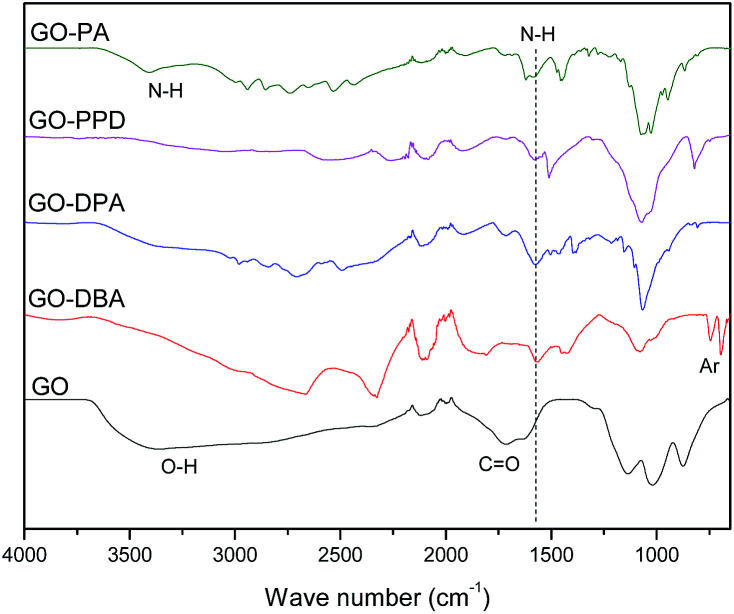
FTIR spectra for GO, GO-DBA, GO-DPA, GO-PPD and GO-PA.

FTIR spectra of GO, GO-PPD and GO-PA, Fig. 5, present a wide O–H stretching band centered at 3600 cm^−1^, related to adsorbed water molecules and also to alcohol and carboxylic acid functional groups. It is also possible to observe the presence of C=O and C=C bond stretching bands at 1715 cm^−1^ and 1618 cm^−1^ respectively, associated to carboxylic, aldehyde and ketone groups and the remaining insaturations of the large areas of sp^2^-hybridized carbons typical of the GO structure.^47^ On the other hand, the functionalized materials exhibit mainly N–H stretching bands in the region from 3400 to 3450 cm^−1^ and at 1550 cm^−1^, indicating a reduction (decrease in oxygen groups) of the graphene oxide structure during the reaction of functionalization with amines in the microwave furnace. Such behavior was also found in the work of Navaee and Salimi,^20^ in which graphene oxide was functionalized with amine groups using ammonia. For the other functionalized GO materials, notably GO-DBA and GO-DPA, Fig. 5, there was a decrease in the intensity of the absorption bands related to the organic ketone, aldehyde and carboxylic acid groups, possibly due to the reduction of the graphene oxide structure. Analogously, N–H stretching bands were observed at around 1550 cm^−1^ and two bands in the 700–850 cm^−1^ region for the structure of the 1,4-disubstituted benzene from the *p*-phenylenediamine structure.^48^

**Fig. 6:**
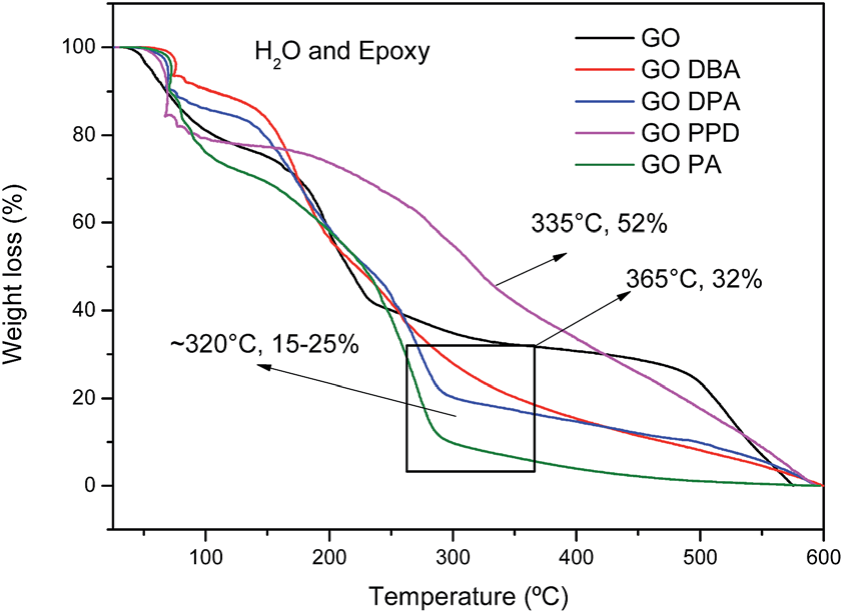
TG curves of GO, GO-DBA, GO-DPA, GO-PPD and GO-PA.

TG curves obtained for the different materials, Fig. 6, exhibit a first weight loss around 100 °C, attributed to the release of water molecules imprisoned in the GO and GO-amines structures and also to the decomposition of epoxy functional groups.^42^ A second weight loss in the temperature range from 160–350 °C is also observed for all materials, related to the elimination of the different oxygenated functions in the carbonic structures, promoting a thermal reduction of the materials, generating thermally reduced graphene oxide.^42^ However, the temperature ranges of these second weight loss events are different. For GO the total chemical reduction occurs at 365 °C, leaving only 32% of the carbon structure mostly without oxygen. For other materials, the reduction ends at around 320 °C, remaining approximately 15 to 25% of initial mass.

TGA results indicate a higher relative weight loss for the amine-functionalized materials than for the non-functionalized graphene oxide, indicating that much of the mass of the functionalized materials may be grafted with amines adsorbed onto the materials, which even after successive washes and drying processes were not removed. However, such behavior has also been observed in the literature for graphene oxide materials highly functionalized with amines or whose amine groups are of high molecular weight.^24^

**Fig. 7:**
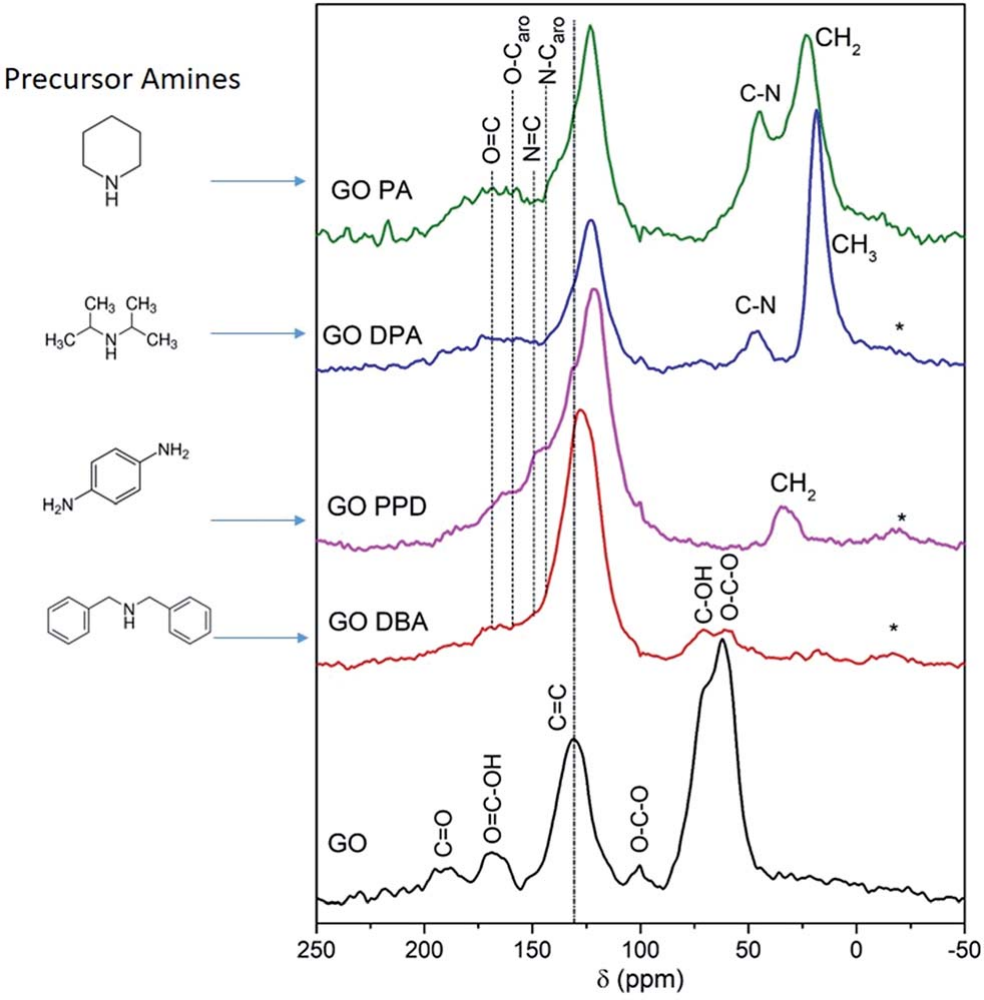
^13^C NMR spectra of GO and GO-amines. The asterisks indicate spinning sidebands.

The solid-state ^13^C NMR spectra recorded for GO and the functionalized materials, Fig. 7, confirm that GO-amines present less oxygen functionalized groups than GO, since the peaks at 60–70 ppm, which are assigned to epoxy and alcohol groups,^38,49^ exhibit much smaller relative intensity in the spectra corresponding to the GO-amine samples when compared to the spectrum obtained for GO. For GO-PPD and GO-PA these peaks are not even detected. The same reasoning can be applied to carbonyl and carboxyl groups, which are abundant in the GO structure and much scarcer in the GO-amines, as shown by the ^13^C NMR spectra. Besides, a peak is observed at 40–50 ppm for GO-PA and GO-DPA samples, associated to the carbons bonded to nitrogen atoms in cyclic or acyclic structures.^38,49–51^ By the relative intensity of the peaks around 20 and 30 ppm, attributed to aliphatic carbons in CH_2_ and CH_3_ groups, especially in the spectra obtained for GO-PA and GO-DPA, one can reiterate the information brought by the TG analyses, which indicate an impregnation of the amines along the structures of reduced graphene oxide. Thus, these findings point to the removal of oxygen-containing groups originally present in GO and, in some cases, their replacement by nitrogen-containing functionalities upon the reactions with the precursor amines.

A further point of interest that can be observed in the spectra shown in Fig. 7 is the chemical shift of the peak associated to sp^2^ carbons (C=C) to lower frequencies (upfield shift) for GO-amine samples, reaching 121 ppm in the case of the GO-PPD sample, in comparison to the GO precursor (where the corresponding chemical shift is 131 ppm). This result is similar to what has been previously reported for GOs functionalized with sulfur-containing groups, where this chemical shift was attributed to an increasing disorder in the basal planes due to the incorporation of the functional groups.^52^ Another likely cause of this upfield chemical shift is the growth of the regions of sp^2^-bonded carbons as a consequence of the already discussed reduction of the oxygen-containing groups. This partial reduction of GO is known to lead to an increase in the effects due to electronic ring currents in the graphene-like areas, which produce an induced magnetic field that contributes to the shielding of ^13^C nuclei and the consequent upfield chemical shift of the ^13^C NMR peak.^42,53^ Following this reasoning, it is expected that a correlation exists between the chemical shift of the peak associated to sp^2^ carbons in the ^13^C NMR spectrum, the relative intensity of the peaks due to epoxy and alcohol groups (at 60–70 ppm)^54^ and the extent of the amine functionalization of each material. In fact, the peak associated to sp^2^ carbons is most upfield shifted in the case of GO-PPD (121 ppm), whose ^13^C NMR spectrum shows no indication of the signals due to the oxygen-containing moieties originally present in the GO structure. This evidence suggests the occurrence of an extensive amine functionalization for this material. On the other hand, for GO-DBA the peak associated to sp^2^ carbons is observed at 128 ppm, which is much closer to the chemical shift observed in the ^13^C NMR spectrum obtained for the parent GO (at 131 ppm); the same spectrum shows significant contributions due to epoxy and alcohol groups (at 60–70 ppm), thus indicating that this material has not been completely functionalized by the amine reaction.

It is also worth noting the high relative intensity of the ^13^C NMR peaks obtained for the GO-PPD sample in the region around 140–150 ppm, which are associated to sp^2^ carbons bonded to nitrogen.^51^ This observation is consistent with the fact that this sample exhibits the highest atomic N content (and the lowest atomic O content) among the studied materials, according to XPS results which will be discussed later. Furthermore, the peak observed at 33 ppm, which is attributed to methylene groups produced as a consequence of the functionalization, is another evidence for the effectiveness of the reaction between GO and the amine in the case of the GO-PPD sample.

**Fig. 8:**
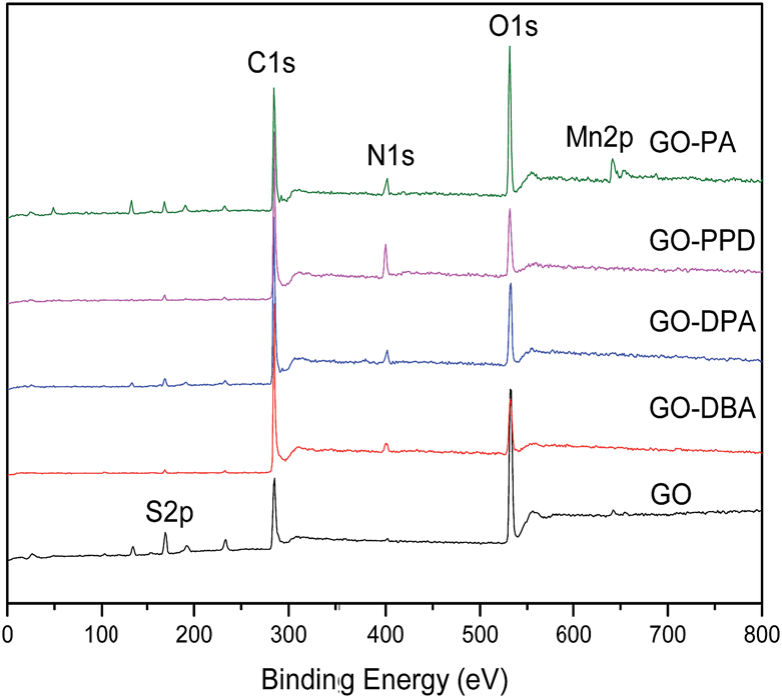
XPS survey spectra of GO and GO-amine samples.

XPS survey spectra, Fig. 8, show the C 1s, O 1s, S 2p and N 1s peaks. The presence of sulfur and manganese as contaminants is due to the use of KMnO_4_ and H_2_SO_4_ in the synthesis of GO. The atomic concentrations are shown in Table 1. As it can be seen, the C atomic concentration of GO-amine samples is higher than the one of GO, whereas the O atomic content follows the opposite trend.

**Table 1:** C, N, O and S atomic concentrations, %, for GO and GO-amines

Sample	% C	% N	% O	% S
GO	50.5	—	41.5	8.0
GO-DBA	81.0	4.0	14.0	1.0
GO-DPA	72.6	4.2	21.0	2.2
GO-PPD	77.0	8.8	13.1	1.1
GO-PA	65.9	5.6	25.8	2.7

This finding is consistent with the partial reduction of GO due to the amine functionalization, as discussed above in connection with the analysis of ^13^C NMR spectra. As also mentioned before, the N atomic concentration is particularly high in GO-PPD, in agreement with the observation of high relative intensity associated with N-containing carbon groups in the ^13^C NMR spectrum of this sample shown in Fig. 7.

**Fig. 9:**
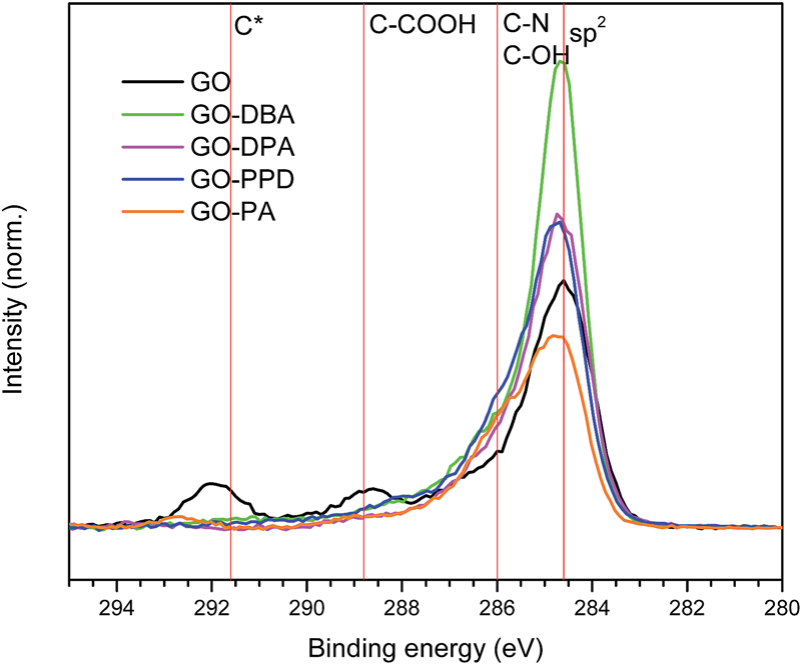
C 1s high resolution XPS spectra obtained for GO, GO-DBA, GO-DPA, GO-PPD and GO-PA.

C 1s high resolution spectra obtained for the GO and GO-amines are shown in Fig. 9. It can be noticed for each spectrum that the carbon atoms are present mostly in the form of aromatic rings in all functionalized samples, which confirms what has been pointed out by the other characterization techniques – *i.e*., that a chemical reduction of the GO structure occurred during the functionalization reactions, with the consequent increase in the content of sp^2^ carbons in the graphene-like layers forming the structure of the GO-amines.

**Fig. 10:**
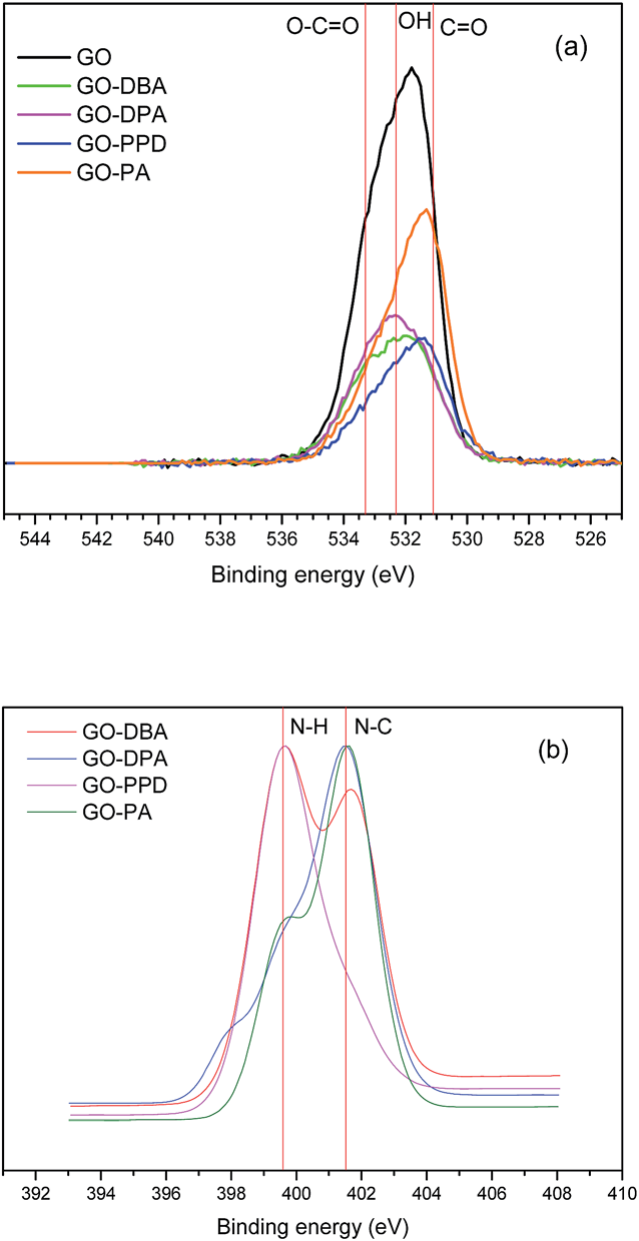
O 1s (a) and N 1s (b) high resolution XPS spectra of GO, GO-DBA, GO-DPA, GO-PPD and GO-PA.

O 1s and N 1s peaks for the GO and GO-amine are shown in Fig. 10a and b. O 1s high resolution spectra show the peaks of O–H (532.7 eV), C=O (531.6 eV) and O–C=O (533.7 eV)^20–22^ in different proportions for each sample. For all materials it can be noticed (Fig. 10b) a bigger contribution of the peak associated to N–C bonds at 401.5 eV, which indicates the formation of a chemical bonding between nitrogen from the amines and the carbon skeleton, therefore, pointing to an actual functionalization of the GO structures. Only in N 1s spectrum for GO-PPD (Fig. 10b) the dominant contribution is due to N–H bonds (399.6 eV), which is expected once its amine structure contains two NH_2_ groups. Some other studies^20,39,47^ have shown similar findings in the XPS analysis of amine-functionalized GO, reinforcing the occurrence of the amine functionalization.

### 3.2 Electrochemical performance

**Fig. 11:**
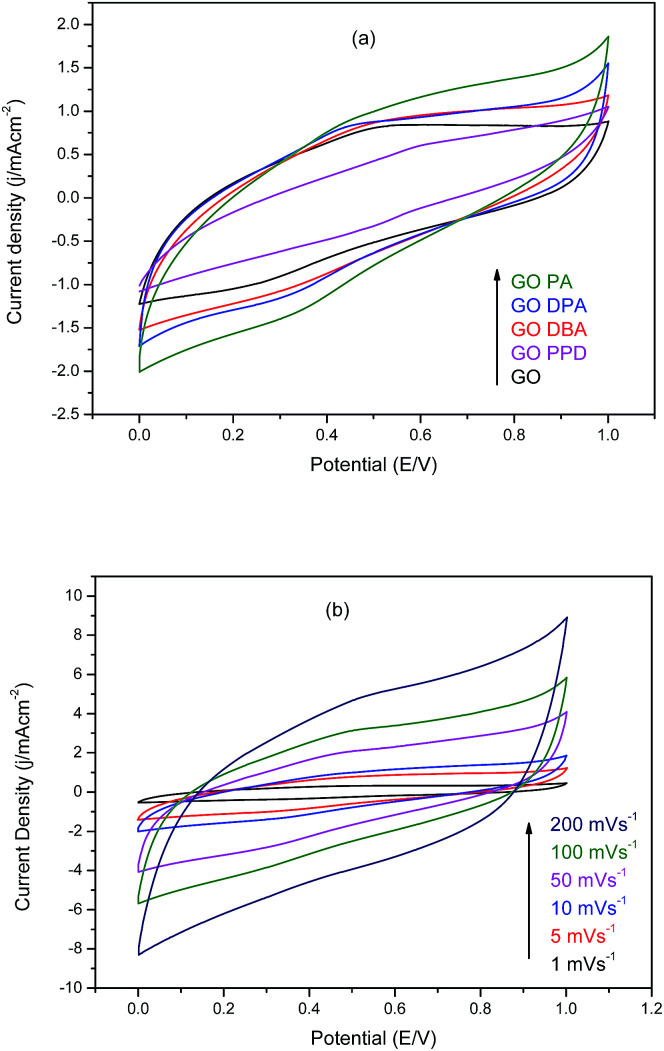
Cyclic voltammograms in 1.0 M H_2_SO_4_ of (a) amine-functionalized GOs at 10 mV s^−1^ and (b) GO-PA at different scan rates.

Cyclic voltammetry (CV) curves obtained for GO and the amine-functionalized GOs performed in 1.0 M H_2_SO_4_ water solution are presented in Fig. 11.

Fig. 11a demonstrates the improvement in electrochemical performance observed for the modified materials when compared to graphene oxide without functionalization, as reported in similar studies,^33–35^ since all amine-GOs reached higher current densities at higher potentials in comparison to GO at 10 mV s^−1^ scan rate. The highest current densities have been achieved by GO-DPA and GO-PA, 1.63 mA cm^−2^ and 1.86 mA cm^−2^ at 1.0 V respectively. Cyclic voltammetry curves obtained for GO-PA at different scan rates, Fig. 11b, confirms the excellent electrochemical behavior of the functionalized materials, reaching current densities of up to 8.9 mA cm^−2^ at 200 mV s^−1^. For these curves, the deviation from rectangularity has also been described in works involving GO amine modifications,^34^ which is associated with the pseudo-capacitance contributions from remaining oxygen-containing groups present in the structures of the functionalized materials.

**Fig. 12:**
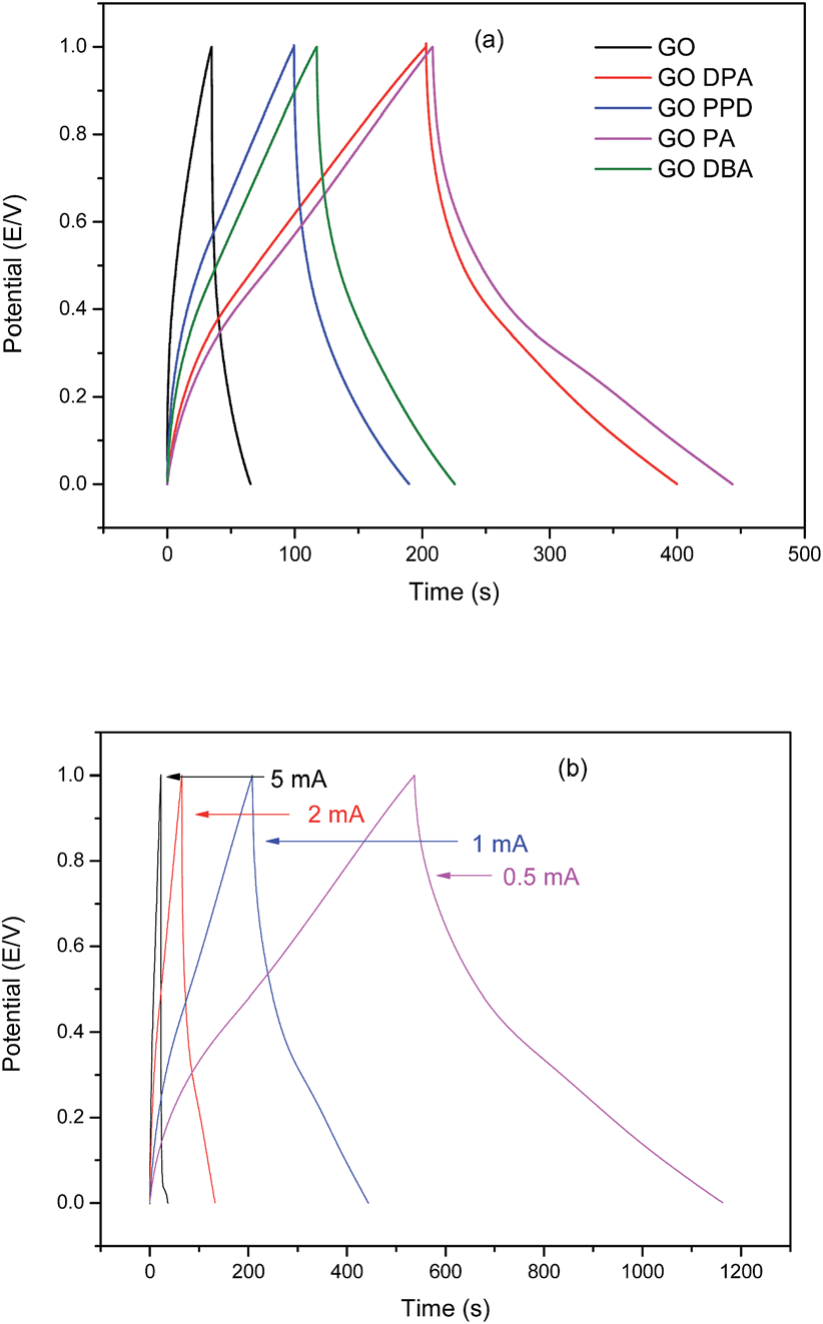
Galvanostatic charge–discharge curves in 1.0 M H_2_SO_4_ for (a) GO and amine-functionalized GO at 1.0 mA cm^−2^ and for (b) GO-DPA at different current densities.

The galvanostatic charge–discharge technique has been applied to determine the specific capacitance for the different materials. The results, presented in Fig. 12, show typical charge–discharge curves for functionalized graphene-based materials.

Fig. 12a confirms the improvement of electrochemical behavior for the modified materials when compared to GO, demonstrated in the CV curves. The two materials which exhibited the highest discharge times at 1 mA cm^−2^ were GO-PA and GO-DPA. The calculated specific capacitances for these materials were 290 F g^−1^ and 260 F g^−1^, respectively, while 205 F g^−1^ for GO-DBA, 174 F g^−1^ for GO-PPD and a much smaller value of 42 F g^−1^ for GO. Similar specific capacitance values have been found for other amine-functionalized graphene oxide materials. Sk and Yue^34^ prepared a layer-by-layer assembly of graphene with *p*-phenylenediamine exhibiting specific capacitances of up to 282.33 F g^−1^ at a discharge current density of 0.75 mA cm^−2^. In the work of Mohammadi *et al.*^32^ and Solonaru and Grigoras^33^ in which amine-functionalized graphene oxide and non-functionalized graphene oxide, respectively, are grafted with different polyanilines the specific capacitance values at 1.0 mA cm^−2^ reached up to 381 F g^−1^ and 650 F g^−1^, respectively. However, in these studies the amine-functionalized GO was not evaluated separately from PANI substrate, thus the high capacitance displayed by their materials cannot be directly considered for comparison with the materials evaluated in the present work, although it is a good indicative of the synergistic effect exhibited by graphene oxide functionalized with amines in supercapacitor applications. Fig. 12b shows the high charge and discharge times exhibited by GO-DPA at lower current densities, approaching 1200 s at 0.5 mA cm^−2^, confirming the increase of specific capacitance at smaller current values. These results are also comparable with those displayed by similar materials in previous works.^33–35^

**Fig. 13:**
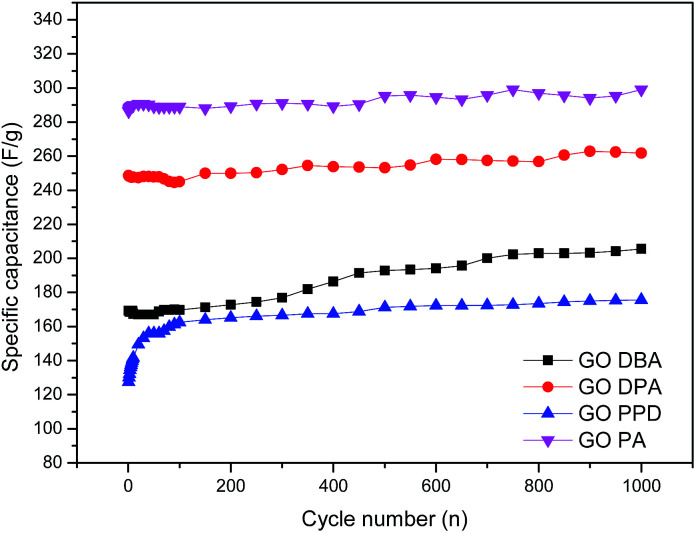
Cyclic stability tests for the amine-functionalized electrodes at 1 mA cm^−2^ discharge current density in aqueous 1.0 M H_2_SO_4_ electrolyte solution.

For better evaluating the electrochemical stability and cycle life efficiency displayed by the different modified electrode materials, continuous charge–discharge cycles were performed at a constant current density of 1 mA cm^−2^, Fig. 13.

As previously indicated by CV and galvanostatic charge–discharge results, the best specific capacitances were obtained for GO-PA and GO-DPA, reaching up to 290 F g^−1^ and 260 F g^−1^, respectively, after 1000 cycles. All materials showed a substantial increase of capacitance with increasing number of cycles; this trend is opposite to the expected behavior of capacitive materials, which should lose performance after continuous cycling.^32–35^ Nonetheless, the increase of capacitance with increasing number of cycles could be easily explained considering the relatively high degree of surface functionalization exhibited mainly by GO-DPA and GO-PA, which was observed in XPS results. The continuous electrochemical charge–discharge processes probably lead to a further surface reduction of the graphene-like structures in the GO amines, increasing their capacitive properties. As described in the work of Wang and collaborators^55^ there is a synergetic interaction between Nitrogen and Sulfur in co-doped graphene structures when applied to supercapacitors. While N is reported to enhance the attraction of ions in graphene layers and enhance the capacitance due to its pseudocapacitive contributions, the sulfur dopant species like sulfone and sulfoxides are described as significant surface modifiers of carbon materials, since the reversible redox reactions they display can also contribute to the pseudocapacitance. Moreover the better electrochemical performance displayed by GO-DPA and GO-PA could be associated with their higher superficial S atomic concentrations, Table 1, when compared to GO-PPD and GO-DBA, whose lower superficial sulfur content might cause a decrease of the N–S synergistic effect in these materials.

**Fig. 14:**
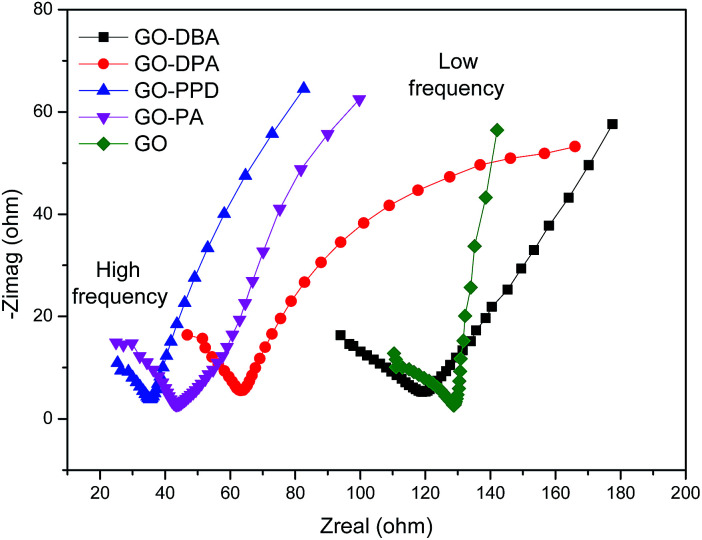
Electrochemical impedance spectroscopy (EIS) of GO and the amine-functionalized electrodes in aqueous 1.0 M H_2_SO_4_ electrolyte solution.

The electrochemical impedance spectroscopy technique was used in order to evaluate conductivity and ion diffusion in the electrochemical systems formed by the amine-functionalized GO electrodes. Nyquist diagrams of impedance for GO and the functionalized electrodes over a frequency range of 100 kHz to 1 Hz are depicted in Fig. 14. In the high frequency region all materials show a semicircle, which has been elsewhere described as a pseudo-transfer resistance and is associated with the porous structure of the electrode.^56^ The semicircles indicate a high sheet resistance for all electrodes (10–15 Ω) which points to a high interfacial charge-transfer resistance that can be associated with a poor conductivity of these materials.^57^ This may be attributed to the adsorption of amines on the graphene surface, as observed from TGA analysis, as well as the presence of remaining oxygen functionalities that can hinder electron transport, like observed for other functionalized graphene electrodes.^58^ In the low frequency region the curves are mostly straight and parallel to the imaginary axis, except for GO-DPA, which indicates a low Warbung resistance, confirming the low ion diffusion resistance typical for capacitive materials.^56^ The lowest resistance values were observed for GO-PPD, GO-PA and GO-DPA.

Finally the results showed that the highest specific capacitance obtained for GO-PA and GO-DPA can be explained by conjunction of three factors: (i) the higher N–S synergistic effect in these materials; (ii) their lower resistance and (iii) the more effective reduction of oxygen-containing functional groups, as previously discussed for ^13^C NMR results. Despite its low resistance GO-PPD shows low specific capacitance, which can be explicated by its low sulfur superficial content. In this case the S–N synergistic effect may play a more important role than the resistance for the specific capacitance.

## 4. Conclusions

In summary, a simple and direct method for the synthesis of amine-functionalized graphene oxide using microwave oven has been proposed. The analysis of the produced materials showed that the four different amines are covalently attached to the graphene oxide structure, even though their interlayer distances have not been increased during the synthesis. All as-synthesized amine-GO materials presented a good electrochemical behaviour, with long life cycle stability and reaching specific capacitance values of up to 290 F g^−1^ and 260 F g^−1^ for GO-PA and GO-DPA samples, respectively, which is in good agreement with other similar reported materials, confirming their potential application as supporting materials in supercapacitors.

## Conflicts of interest

There are no conflicts to declare.

## Supplementary Material
